# Susceptibility of Ferret and Cat to Porcine Deltacoronavirus: Evidence of Infection in Ferrets But Not Cats

**DOI:** 10.1155/tbed/9997711

**Published:** 2025-06-10

**Authors:** Shuhuai Meng, Renqiang Liu, Hao Zhang, Guangli Hu, Lei Shuai, Huijuan Guo, Yijing Dang, Yongchang Cao, Zhigao Bu, Zhiyuan Wen

**Affiliations:** ^1^State Key Laboratory for Animal Disease Control and Prevention, Harbin Veterinary Research Institute, Chinese Academy of Agricultural Sciences, Harbin 150069, Heilongjiang, China; ^2^State Key Laboratory of Biocontrol, School of Life Sciences, Sun Yat-sen University, Guangzhou 510006, Guangdong, China; ^3^Jiangsu Co-innovation Center for Prevention and Control of Important Animal Infectious Diseases and Zoonoses, Yangzhou University, Yangzhou 225009, Jiangsu, China

**Keywords:** cats, ferrets, interspecies transmission, porcine deltacoronavirus (PDCoV)

## Abstract

Coronaviruses (CoVs) cause gastrointestinal and respiratory disorders and have a wide host range. Porcine deltacoronavirus (PDCoV) is an enteropathogenic CoV and a member of the *Deltacoronavirus* (δ-CoV) genus and was discovered in 2012. With a high fatality rate, PDCoV is primarily responsible for severe diarrhea in pigs, especially in newborn piglets, and has been reported worldwide since the first outbreak in 2014. PDCoV is confirmed as a zoonotic virus, and one of the few CoVs currently known to infect both birds and mammals, including humans. Infection studies have demonstrated the susceptibility of mice and calves to PDCoV, but little is known about its transmission in other mammals. Before it was reported in pigs, deltacoronavruses had been reported in ferret badgers and Asian leopard cats in China in 2007. This implies the possibility that wild mustelids and feline species contribute to the virus's spread. In this study, we investigated the susceptibility of ferrets and domestic cats to PDCoV. The results revealed that ferrets can be infected with PDCoV, while cats cannot. In ferrets, viral RNA was detected in the intestines, parenchymal organs, and feces. Histopathological analysis showed no visible lesions in the intestine of infected ferret and cat. Neither infected ferrets nor cats exhibited any clinical signs of diarrhea, but seroconversion occurred in ferrets 14 days post-inoculation (dpi). In brief, ferrets may play a significant role in the interspecies transmission of PDCoV. Our study expands the known host range of PDCoV and provides valuable insights into the interspecies transmission of the virus.

## 1. Introduction

Coronaviruses (CoVs) are enveloped, single-stranded, positive-sense RNA viruses belonging to the order *Nidovirales* and family *Coronaviridae* [1]. They can cause both respiratory and gastrointestinal diseases in a broad host range. To date, many worldwide pandemics have been caused by CoVs, including severe acute respiratory syndrome CoV (SARS-CoV), Middle East respiratory syndrome CoV (MERS-CoV), and SARS-CoV-2 [2]. These outbreaks have demonstrated that CoVs have the potential to spread significantly across species, leading to major risks and losses to public health. CoVs are classified into four genera: *Alphacoronavirus* (*α*-CoV), *Betacoronavirus* (*β*-CoV), *Gammacoronavirus* (*γ*-CoV), and *Deltacoronavirus* (δ-CoV) [3]. δ-CoV, which mainly infects birds, was initially discovered in Asian leopard cats and Chinese ferret badgers in southern Chinese wildlife markets [4]. From 2007 to 2012, several novel δ-CoVs were identified in Hong Kong through molecular virological studies. These viruses were shown to be capable of infecting both birds and mammals, signifying the initial recognition of porcine deltacoronavirus (PDCoV) [5, 6].

PDCoV is a novel porcine enteric pathogenic CoV that causes severe watery diarrhea and intestinal lesions, particularly in newborn piglets [7]. Since the first PDCoV outbreak in a pig farm in Ohio, USA, in 2014 [8], the virus has spread to several countries in North America and Asia [9–12], causing the swine industry to suffer large financial losses. Recently, PDCoV was isolated from the plasma samples of three Haitian children with acute febrile illness, which emphasizes both the possibility that it poses a health risk to humans and the necessity of determining how it spreads between different species [13].

Surveillance of wildlife and laboratory infections has demonstrated the broad host range of PDCoV, including wild birds [14], chickens [15], and turkeys [16], as well as mammals, including mice [17, 18] and calves [19], which have shown susceptibility to the virus. This suggests that PDCoV has significant interspecies transmission potential. Although emerging evidence indicates that PDCoV likely originated from a sparrow HKU17-like CoV [20], its host range has not yet been fully defined, which indicates the need for susceptibility studies in various animals. Ferret and cat are known hosts for several CoVs [21, 22]; recent studies have shown that ferret and cat are also susceptible to SARS-CoV-2 [23]. Ferrets are commonly used as mammalian models in virology research [24, 25], and both ferret and cat are widely kept as companion animals with close associations with human habitats [26, 27]. Moreover, the *S* gene of PDCoV shares over 99.8% nucleotide similarity with δ-CoVs isolated from Asian leopard cats and Chinese ferret badgers [6]. Therefore, it is crucial to understand the susceptibility of these animals to PDCoV and assess their roles in virus transmission and evolution. To our knowledge, no studies have yet reported the susceptibility of ferret and cat to PDCoV. In this study, we show that ferrets are susceptible to PDCoV infection while cats are not. These findings broaden the understood host range of PDCoV infection and indicate that small mustelids, particularly ferrets, could serve as significant hosts for the virus.

## 2. Materials and Methods

### 2.1. Virus and Cells

The PDCoV CHN-GD-2016 (GenBank: MF280390.1) [7] strain was serially passaged and cultured in porcine proximal tubular cells (LLC-PK), which were grown in Dulbecco's Modified Eagle Medium (DMEM) (Sigma–Aldrich, USA), supplemented with 10% fetal bovine serum (ExCell, China). The viral titers were assessed through the 50% tissue culture infectious dose (TCID_50_) method in LLC-PK cells.

### 2.2. Animals

Clean (CL) ferrets and domestic cats were obtained from the Harbin Veterinary Research Institute. According to the Chinese National Standard GB 14922.2-2011, the definition of CL animal is as follows [28]: In addition to the pathogens excluded in conventional animals, these animals are further free from pathogens that pose significant risks to animal health or substantially interfere with scientific research outcomes. Their breeding and testing adhered to the national standards in the Pharmacopoeia of the People's Republic of China (2015 edition).

All animals were kept in isolation in a controlled environment at the Animal Experimental Center of the Harbin Veterinary Research Institute, Chinese Academy of Agricultural Sciences, where they received sterile food and water. The experimental protocol and housing conditions were approved by the Animal Ethics Committee of the Harbin Veterinary Research Institute, Chinese Academy of Agricultural Sciences (Approval No: 230310-02-GR, 240314-01-GR).

### 2.3. Virus Inoculation

The selected experimental animals included 2-month-old ferrets (*n* = 40) and subadult cats aged 6–9 months (*n* = 40). The experiment was designed with two groups: the inoculation group (*n* = 25) and the control group (*n* = 15), with animals randomly assigned to each group. Animals from different groups were housed in separate isolation units. Prior to inoculation, all ferret and cat tested seronegative for PDCoV antibodies by enzyme-linked immunosorbent assay (ELISA), and their fecal samples were negative for viral RNA by quantitative real-time RT-PCR (qRT-PCR).

Considering the differences in body weight between ferret and cat, the dosage of virus inoculation was adjusted accordingly. The inoculation group received an oral dose of DMEM containing 1 × 10^6^ TCID_50_ of PDCoV, with volumes of 300 μL for ferrets and 2 mL for cats. The control group was inoculated with the same volume of blank DMEM.

Animals were observed throughout the day for defecation and fecal morphology to assign scores. The scoring criteria were as follows: 0 = normal feces, 1 = soft but formed feces, 2 = semi-liquid feces, 3 = watery diarrhea, with a score of 2 or above considered as diarrhea.

### 2.4. Sample Collection

At 3, 5, 7, 10, and 14 dpi, rectal swabs were collected by inserting a cotton swab ~5 cm into the rectum. After collection, the swabs were immediately placed in 1.5 mL centrifuge tubes containing 1 mL of DMEM and kept on ice until reaching the laboratory. The centrifuge tubes were vigorously vortexed for 10 s and then centrifuged at 1800 × *g* for 5 min at 4°C. The supernatant was aspirated using a pipette for subsequent viral RNA extraction.

On the same days as the rectal swab collection, five animals from the inoculation group and three animals from the control group were randomly selected. The animals were euthanized, followed by dissection. Euthanasia was performed by intramuscular injection of Zoletil 50 (Virbac, France) at a dose of 15 mg/kg for anesthesia. Once deep anesthesia was confirmed by the absence of reflexes and loss of consciousness, potassium chloride solution was administered via intracardiac injection at a dose of 150 mg/kg to induce cardiac arrest. After the cessation of vital signs, organ sample collection was performed. The euthanasia procedure was conducted in accordance with the AVMA Guidelines for the Euthanasia of Animals: 2020 Edition. To ensure the quality of tissue samples for subsequent histopathological analysis, exsanguination was carried out after death confirmation. Tissue samples were collected from the heart, liver, spleen, lungs, kidneys, duodenum, pancreas, jejunum, ileum, colon (in ferrets), cecum (in cats), and rectum. Fresh samples from each organ were prepared for RNA extraction for subsequent nucleic acid testing, while additional samples were collected for pathological examination.

On day 14 dpi, three animals were randomly selected from both the inoculation and control groups for blood collection, which was processed into serum for serological testing.

### 2.5. Virus RNA Extraction, qRT-PCR, and RT-PCR

Approximately 100 mg of fresh organ sample obtained from dissection was placed in a grinding tube containing 1 mL of phosphate-buffered saline (PBS) with garnet particles and ceramic beads. The grinding tube was then placed in a FastPrep-24 5G homogenizer (MP Biomedicals, USA) to create a suspension. The processed samples were centrifuged at 2000 × *g* for 10 min at 4°C to separate the supernatant from the grinding material. Subsequently, 200 μL of the supernatant was aspirated and added to Virus DNA/RNA Extraction Kit 2.0 (Vazyme, China), which was then placed in an automatic nucleic acid extractor (Vazyme, China), using a preset program to extract viral RNA from the samples. The obtained viral RNA was reverse transcribed using HiScript III 1st Strand cDNA Synthesis Kit (Vazyme, China) to yield total complementary DNA (cDNA), which was used for qRT-PCR.

The primers and probes required for the qRT-PCR experiment were designed to target the PDCoV *M* gene. The sequences are as follows: FPD-M: ATCGACCACATGGCTCCAA, RPD-M: CAGCTCTTGCCCATGTAGCTT, PROBE-M:FAM-CACACCAGTCGTTAAGCATGGCAAGCT-BHQ. The amplified fragment size is 72 bp.

The primers were designed to target the PDCoV *S* gene with the following sequences: SF:CAACCGTCTTGAGGAAGTAGAG, SR: TCAACGGTGAGGTTGAGAATAG. The amplified fragment size is 609 bp. All primers and probes were designed based on the genome sequence of the CHN-GD-2016 strain.

qRT-PCR was conducted using a QuantStudio 5 fluorescence quantitative PCR system (Applied Biosystems, USA), with data analysis performed through the instrument's integrated software. The sensitivity threshold for qRT-PCR was determined to be 4.6 log_10_ copies/g (organ sample) and 4.6 log_10_ copies/mL (fecal supernatant). Positive controls consisted of a 10-fold dilution series of standard plasmids for each RT-PCR and qRT-PCR reaction, while negative controls included samples from mock infections and PBS.

### 2.6. Histopathology

Tissue samples from ferret and cat, including heart, liver, spleen, lungs, kidneys, duodenum, pancreas, jejunum, ileum, colon (ferrets), cecum (cats), and rectum, were collected and preserved in 4% paraformaldehyde (PFA) at room temperature for a week. Following fixation, the samples were embedded in paraffin, sliced into sections, stained with hematoxylin and eosin (H&E), and subsequently analyzed using an light microscope.

### 2.7. ELISA for Detection of PDCoV-Specific Antibodies in Serum

An indirect ELISA was utilized to detect specific IgG antibodies against PDCoV in serum samples. About 5 µg of inactivated PDCoV was coated onto a 96-well plate using 0.05 M carbonate buffer solution (CBS) at pH 9.6, with 100 µL of the solution per well, and incubated overnight at 4°C. After washing the plate three times with PBS containing 0.05% Tween-20 (PBST), a 5% skim milk solution was added for blocking at 37°C for 2 h, followed by three washes with PBST. Next, 10 µL of serum from ferret and cat was serially diluted twofold in 5% skim milk. The 100 µL diluted serum–milk mixture was added sequentially to the virus-coated 96-well plate and incubated at 37°C for 1 h. The plate was then washed three times with PBST. Antibodies conjugated with horseradish peroxidase were added (goat anti-ferret IgG, Kirkegaard & Perry Laboratories, USA; Rabbit anti-cat, Biodragon, China), followed by a 1-h incubation at 37°C and three additional washes with PBST. Tetramethylbenzidine (TMB) substrate was then added for color development, and the reaction was allowed to proceed for 15 min at 37°C. Finally, 2 M sulfuric acid was added to each well to terminate the color reaction. The absorbance was measured at 450 nm using ELx808IU plate reader (BioTek, USA).

### 2.8. Sequencing of PDCoV *S* Gene in Ferrets

Primers were designed to amplify the full-length *S* gene based on the sequence of CHN-GD-2016, specifically PDS-F and PDS-R (sequences: PDS-F: ATGCAGAGAGCTCTAT, PDS-R: CTACCATTCCTTAAACTTA). Positive samples from ferrets were selected, and total RNA was extracted using the previously described method, followed by reverse transcription to obtain cDNA, which was then used for *S* gene amplification. The PCR reaction was conducted using KOD One PCR Master Mix DNA polymerase (TOYOBO, Japan) to amplify the *S* gene, with the following PCR program: 98°C for 5 min for initial denaturation, followed by 40 cycles of 98°C for 20 s for denaturation, 55°C for 15 s for annealing, and 68°C for 4 min for extension, with a final extension at 68°C for 10 min. Ultimately, the full-length *S* gene of PDCoV was amplified, cloned, and sequenced from the ferret samples.

### 2.9. Statistical Analysis

Statistical analyses were performed using the one-way ANOVA function of GraphPad Prism 8.0 to calculate *p* values. The results are presented as mean values ± standard errors of the mean (SEM).

## 3. Results

### 3.1. Viral Shedding Was Detected in Ferret Intestines With No Diarrhea Observed in Ferrets or Cats

**Figure 1:**
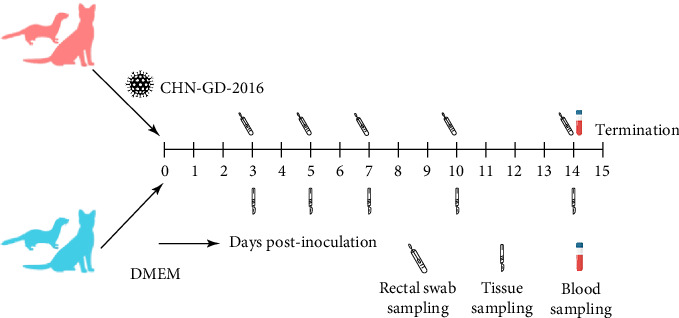
Experimental procedure. Due to the differences in body weight between ferret and cat, the dosage of virus inoculation was adjusted accordingly. For all animals in inoculated groups, 6 log_10_ TCID_50_ PDCoV CHN-GD-2016 was administered. The cats were 6–9 months old, and the ferrets were 2 months old. Depending on their size, the cats received a 2 mL oral inoculation, whereas the ferrets received 0.3 mL.

**Figure 2:**
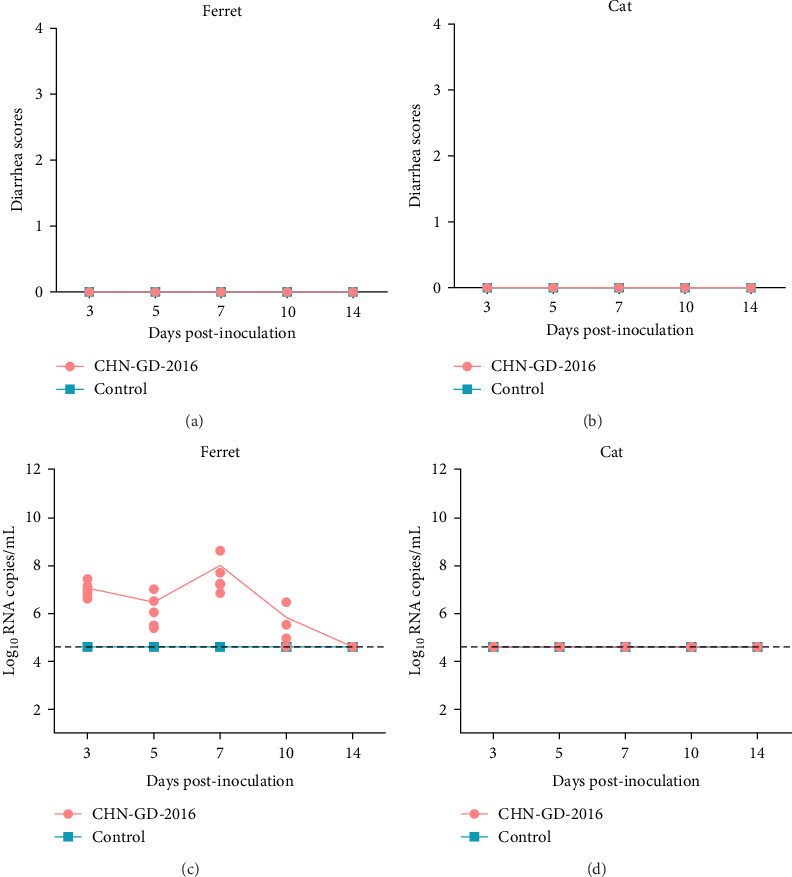
Diarrhea scores (A and B) and fecal virus RNA shedding (C and D) in ferret and cat inoculated with porcine deltacoronavirus (PDCoV) strain CHN-GD-2016. Diarrhea scores for ferrets (A) and cats (B) were monitored daily postinoculation. Viral RNA shedding in ferrets (C) and cats (D) feces was determined using quantitative real-time RT-PCR (qRT-PCR). The qRT-PCR detection limit for virus RNA in fecal samples was 4.6 log_10_ copies/mL of PDCoV, as shown by the dotted lines in the figure.

About 2-month-old ferrets and subadult domestic cats aged 6–9 months were selected for the experiment and administered the CHN-GD-2016 strain orally. Diarrhea signs were assessed concurrently with the collection of rectal swab samples at 3, 5, 7, 10, and 14 dpi (Figure 1). Viral RNA was detected in the rectal swabs of ferrets at 3, 5, 7 and 10 dpi, with the highest viral levels observed at 7 dpi (around 8 log_10_ RNA copies/mL) (Figure 2C). In contrast, no viral RNA was detected in the swab samples from cats throughout the experimental period (Figure 2D). Neither cats nor ferrets exhibited any signs of diarrhea (Figure 2A,B). Additionally, no viral RNA was detected in the swabs from the control group animals, which also did not display any signs of diarrhea.

### 3.2. Viral Genomes Detected in the Intestine and Other Parenchymal Organs of Inoculated Ferrets

**Figure 3:**
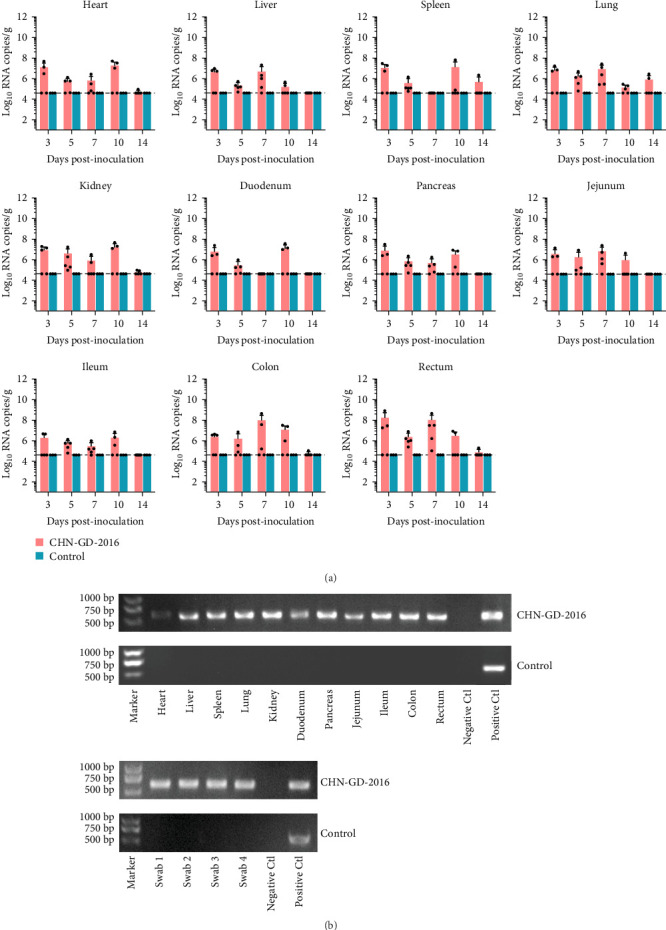
Distribution of virus RNA in heart, liver, spleen, lungs, kidneys, duodenum, pancreas, jejunum, ileum, colon, and rectum of ferrets using qRT-PCR (A) and specific amplification of the viral *S* gene in tissues and rectal swabs (B) detected by RT-PCR. The distribution and dynamics of PDCoV in various organs of inoculated and control ferrets were analyzed using qRT-PCR at 3, 5, 7, 10, and 14 dpi (A). The specific amplification of the PDCoV *S* gene fragment in organ samples from inoculated groups was tested positive by qRT-PCR at 10 dpi via RT-PCR (B). As for rectal swabs, swab1-4 were qRT-PCR positive samples from 3, 5, 7, and 10 dpi, respectively. The qRT-PCR detection limit for virus RNA in organ samples was 4.6 log_10_ copies/g of PDCoV, as shown by the dotted lines in the figure.

**Figure 4:**
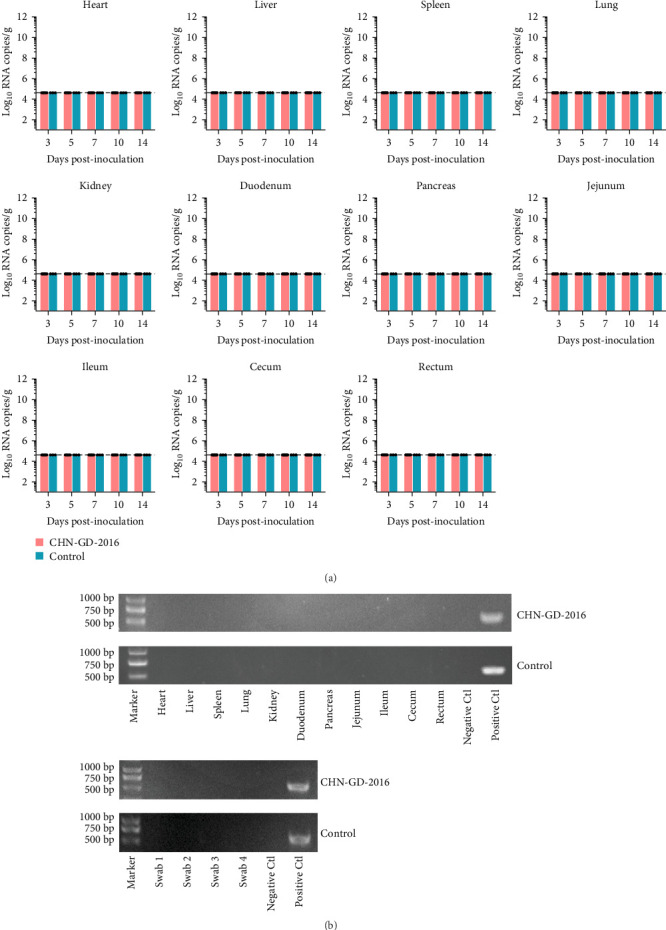
Distribution of virus RNA in heart, liver, spleen, lungs, kidneys, duodenum, pancreas, jejunum, ileum, cecum, and rectum of cats using qRT-PCR (A) and specific amplification of the viral *S* gene in tissues and rectal swabs (B) detected by RT-PCR. The distribution and dynamics of PDCoV in various organs of inoculated and control cats were analyzed using qRT-PCR at 3, 5, 7, 10, and 14 dpi (A). The specific amplification of the PDCoV *S* gene fragment in organ samples from inoculated groups was tested positive by qRT-PCR at 10 dpi via RT-PCR (B). As for rectal swabs, swab1-4 were random samples from 3, 5, 7, and 10 dpi, respectively. The qRT-PCR detection limit for virus RNA in organ samples was 4.6 log_10_ copies/g of PDCoV, as shown by the dotted lines in the figure.

In ferret, organ samples were collected at 3, 5, 7, 10, and 14 dpi and analyzed using qRT-PCR to investigate the temporal variation of viral levels in the animals and the organ tropism. The results indicated that viral RNA copies were detectable in all organs of the inoculated ferrets (Figure 3A), suggesting a systemic infection. The dynamic distribution of viral copies exhibited a fluctuating trend. High levels of viral load were detected at 3 dpi, after which it declined and then increased again at 7 or 10 dpi. (ranging from ~7 to 8 log_10_ RNA copies/g), and then dropped to the lowest levels by 14 dpi (Figure 3A). Since qRT-PCR detected positive results in all 10 dpi organ samples, those samples were selected for further analysis using RT-PCR to assess specific amplification. Amplification of the *S* gene fragment revealed products in the ferret samples that corresponded to the designed primers, with a fragment size of 609 bp (Figure 3B); amplified fragments were subsequently Sanger sequenced, and the results showed that their genetic sequences matched the *S* gene sequence of CHN-GD-2016. This result confirms the presence of PDCoV in these samples. In cats, no viral RNA was detected (Figure 4).

### 3.3. Seroconversion in Inoculated Ferret and Cat

**Figure 5:**
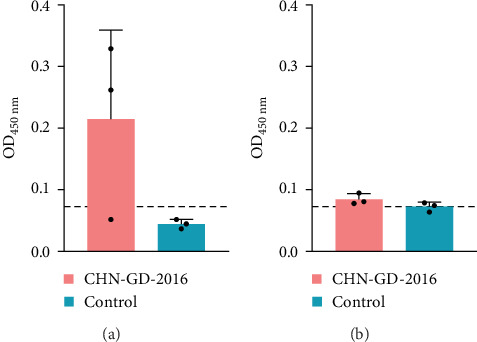
IgG antibody levels in ferret serum samples (A) and cat serum sample (B) at 14 dpi. The IgG antibody levels against PDCoV in ferrets (A) and cats (B) were measured using serum samples collected at 14 dpi. Data are presented as the mean of three replicates with standard deviation, and the shape of the points represents the values of individual samples. The dotted line indicates the cutoff line.

To further confirm whether the animals were infected with PDCoV, the levels of anti-PDCoV IgG antibodies in serum samples were assessed using an indirect ELISA method (Figure 5). At 14 dpi, most of the ferrets (two out of three ferrets of the inoculation group) showed an increase in serum IgG antibody levels, indicating a positive response and suggesting a mild infection, whereas the cats did not show a significant increase in IgG antibody levels, which indicates that cats did not induce robust antibody response against the virus. This observation suggests that PDCoV did not develop an effective infection in the inoculated cats.

### 3.4. Pathological Lesions in the Intestines of Inoculated Ferrets

**Figure 6:**
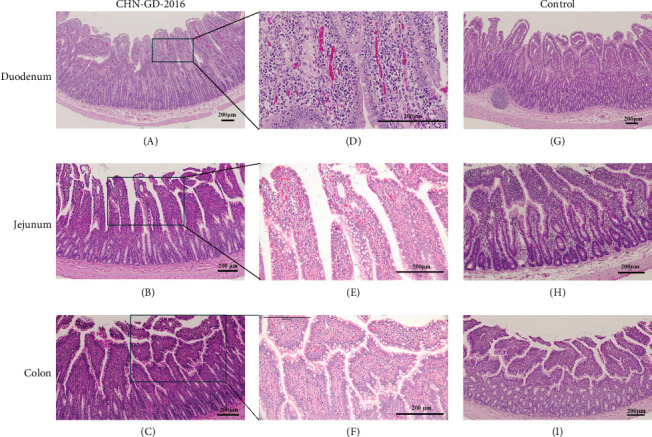
Pathology of tissue sections from ferrets inoculated with PDCoV. Tissue sections from CL ferrets inoculated with PDCoV CHN-GD-2016 were collected at 10 dpi (A–F), along with tissues from the control group (G–I), including duodenum (A, D, G), jejunum (B, E, H), and colon (C, F, I). After fixation, the tissues were stained for observation. (D–F) represent magnified views of (A–C), respectively. Staining was performed using the hematoxylin and eosin method.

In the intestinal tissues of inoculated ferrets, mild lesions were observed. Based on qRT-PCR results indicating positive viral detection and high viral RNA copy numbers at 10 dpi, we selected intestinal samples for H&E staining. Although inflammatory cell infiltration in the lamina propria and mucosa, as well as degeneration of mucosal epithelial cells, were noted in the duodenum, jejunum, and colon of the inoculated group. However, there were no signs of significant intestinal lesions, such as reduced, atrophied, or shortened intestinal villi. Additionally, the rectum showed no evident damage. Notably, these findings are consistent with the absence of diarrhea in the inoculated ferrets (Figure 6).

### 3.5. Sequencing of *S* Gene Amplified From Inoculated Ferrets

Samples of heart, liver, spleen, lung, kidney, jejunum, ileum, colon, rectum, and anal swabs were collected from ferrets that tested positive for PDCoV at 10 dpi. These samples were utilized to amplify the *S* gene of PDCoV using targeted primers, aiming to assess whether any mutations had arisen during the experimental procedures. Subsequent sequencing of the amplified gene and its comparison with the original strain's sequence demonstrated that the *S* gene sequence from the ferret samples was identical to that of CHN-GD-2016. The sequencing data indicated that the *S* gene comprised 3480 nucleotides, encoding a protein consisting of 1160 amino acids. Alignment of the sequences revealed no mutations in the *S* gene of PDCoV throughout the experiment, suggesting that viral passage in ferrets did not induce any alterations in the virus's *S* gene (File S1).

## 4. Discussion

The coronaviruses responsible for global pandemics including SARS-CoV, MERS-CoV, and SARS-CoV-2, frequently undergo mutations as a result of interspecies transmission, which can result in the emergence of novel infectious diseases that pose a serious threat to public health. Since PDCoV is one of the few CoVs known to infect both birds and mammals, knowledge of its host range is crucial for determining and reducing the risk of interspecies transmission [29].

Our investigation into the susceptibility of ferret and cat to PDCoV revealed additional details about the pathogenesis and transmission dynamics of this virus. However, this study does have certain limitations in its experimental design. In susceptibility studies, animal age is a critical variable. Initially, we planned to uniformly use juvenile animals. Nonetheless, our investigation into PDCoV susceptibility in cats prioritized the use of 6–9-month-old subadult cats over juvenile cats due to both scientific and ethical considerations. Juvenile cats, particularly during the weaning phase, exhibit biological vulnerabilities, including immature immune systems, underdeveloped gastrointestinal barriers, and transitional intestinal morphology with unstable digestive enzyme activity. These features render them prone to nonspecific diarrhea triggered by environmental stressors such as transportation, acoustic disturbances, or cohousing with unfamiliar littermates [30–32]. These risks are further exacerbated by the absence of maternally derived antibodies in laboratory settings. Additionally, juvenile cats demonstrate a heightened sensitivity to procedural stressors (e.g., restraint, sample collection) and environmental stimuli (e.g., light, sound), which may confound experimental outcomes through stress-induced gastrointestinal dysfunction or even fatal complications, as observed in previous studies. In contrast, subadult cats offer superior experimental validity owing to their stabilized gastrointestinal function, established vaccination history and enhanced stress tolerance. This minimizes age-related confounding variables while aligning with animal welfare standards. Moreover, this approach is supported by our laboratory's previous CoV research, in which subadult cats yielded results comparable to those from juvenile cats [23], thereby ensuring both methodological rigor and ethical compliance.

Despite the early detection of viral RNA in rectal swabs in our study, no clinical signs of diarrhea were observed in ferrets. Although overall levels were low, the virus showed broad tissue tropism in ferrets, with different viral load distributions between parenchymal and digestive organs. Interestingly, the absence of diarrhea and the failure to detect viral copies in organ samples and rectal swabs of cats indicated that the inoculated cats were not successfully infected. The aforementioned findings suggest that ferret and cat have different susceptibilities to PDCoV.

Serological and histopathological analyses further corroborated the aforementioned results, showing only minor serological alterations and limited tissue damage in ferrets.

Although the inoculated ferrets in this study showed no clinical signs of diarrhea, the high viral RNA loads detected in intestinal tissues (peaking at ~7–8 Log_10_ RNA copies/g) and prolonged fecal viral shedding (up to 10 dpi) suggest potential transmission risks. Asymptomatic carriers are known to facilitate viral spread in other coronaviral systems, such as SARS-CoV-2 transmission by asymptomatic humans [33] and the transmission of the virus from asymptomatic hamsters to humans, as well as its spread among North American white-tailed deer populations [34, 35]. Moreover, although ferrets exhibited a relatively mild level of infection in the experimental setting, higher viral loads in natural environments, along with host selection pressures, may drive viral mutations in asymptomatic ferrets, potentially leading to the emergence of novel infectious diseases with unforeseen consequences.

Previous studies have demonstrated that chicks, turkeys, chicken embryos, as well as mammals like mice and calves [15, 16, 18, 19] can be infected with PDCoV, which highlights its strong inter-species potential. This underscores the necessity of investigating the susceptibility of a broader range of mammals to PDCoV. Ferret and cat are known to be susceptible to several CoVs, including ferret enteric CoV (FRECV), ferret systemic CoV (FRSCV), feline enteric CoV (FECV), and feline infectious peritonitis virus (FIPV) [36], as well as SARS-CoV-2 [23]. Molecular virological studies, including origin tracing and sequences comparison of CoVs like SARS-CoV, MERS-CoV, and SARS-CoV-2, have confirmed that mutation and recombination of the viral genome during interspecies transmission among mammals are key factors in the emergence of new CoVs [37]. The origin of canine CoV (CCoV) is believed to be related to feline CoV (FCoV) [38], while porcine transmissible gastroenteritis virus (TGEV) is originated from cross-species transmission of CCoV [38, 39]. Although PDCoV does not belong to the same genus as CoVs mentioned above, its transmission from birds to pigs and its high genetic similarity to DCoVs previously detected in Asian leopard cats and Chinese ferret-badgers suggest that PDCoV has significant potential for inter-species transmission among mammals. Our study demonstrated that ferrets are susceptible to PDCoV, whereas cats are not. In summary, since ferret and cat are companion animals that are intimately linked to human life, awareness of their roles in the spread of PDCoV is essential to the successful prevention and management of the viruses.

## 5. Conclusion

Our study concludes that ferrets are susceptible to PDCoV, as evidenced by mild pathological changes and antibody responses in the absence of clinical signs of diarrhea. Conversely, cats exhibited no detectable viral RNA levels or seroconversion, nor did they show any susceptibility to PDCoV infection. These results expand the known host range of PDCoV and imply that ferrets might contribute to the virus's interspecies transmission. Consequently, it is imperative to further investigate PDCoV transmission dynamics and host susceptibility in greater detail to inform effective virus control and prevention strategies.

## Data Availability

The data that support the findings of this study are available from the corresponding author upon reasonable request.

## Supporting Information

Supporting Information File S1. *S* gene sequences of PDCoV CHN-GD-2016 strain in cell culture and ferret ileum tissue.
