# Supplementary material for: Susceptibility of Ferret and Cat to Porcine Deltacoronavirus: Evidence of Infection in Ferrets But Not Cats
Source: Transbound Emerg Dis. 2025 Jun 10;2025:9997711. doi: 10.1155/tbed/9997711 (PMC12173537; doi:10.1155/tbed/9997711)
Supplement: Supporting Information — File S1. S gene sequences of PDCoV CHN-GD-2016 strain in cell culture and ferret ileum tissue. [file 9997711.f1.pdf]

***S* Gene Sequences of PDCoV CHN-GD-2016 strain in Cell Culture and Ferret Ileum Tissue.**

Sequencing of all the amplified *S* gene samples produced consistent results. One ferret (10 dpi) in ileum tissue is shown here. In the ferret samples, the *S* gene sequence matched that of CHN-GD-2016.

S gene sequence of CHN-GD-2016

ATGCAGAGAGCTCTATTGATTATGACCTTACTTTGTCTCGTTCGAGCAAAGTTTGCTGAT
GATCTACTCGATTTGCTCACCTTCCCGGGTGCACATCGCTTCTTACATAAACTCACGAGT
AATTCCAGCAGTCTCTACTCGCGGGCTAATAACTTTGATGTTGGCGTTCTTCCTGGCTA
CCCCACTAAGAACGTTAACCTCTTCTCACCACTTACTAACTCTACTTTGCCAATTAATGG
CCTTCATCGGAGTTACCAACCTCTTATGCTGAATTGTCTTACTAAAATAACTAACCACAC
TCTCAGCATGTATCTCCTACCTAGTGAGATACAAACTTATAGCTGCGGCGGTGCCATGG
TTAAATACCAGACACATGATGCAGTTCGTATCATTTTAGACCTCACTGTCACTGACCAC
ATCTCTGTTGAAGTCGTTGGCCAACGTGGTGAAAATTATGTGTTTGTTTGTAGTGAGCA
GTTTAACTATACCACTGCATTACCCAACTCTACCTTCTTCTCACTTAATTCTGAGCTTTAT
TGCTTTATTAATAACACCTACTTAGGTATTCTTCCACCTGATTTAACTGACTTTACGGTCT
ATCGTACTGGGCAGTTTTATGCTAATGGTTACCTTTTAGGTACTTTACCTATTACGGTTAA
CTATGTAAGGTTGTATCGGGGTCATTTGTCGGCCAATAGTGCCCACTTTGCCCTTGCAA
ACCTAACCGATACACTCATAACACTTACCAATACTACTATATCGCAAATCACTTATTGTG
ATAAGTCAGTAGTTGATTCAATAGCATGCCAGCGCTCTTCTCACGAAGTGGAGGATGGG
TTTTACTCCGACCCTAAATCTGCCGTTAGAGCTAGGCAACGTACTATTGTTACACTACCT
AAGCTCCCTGAGCTTGAAGTAGTGCAGTTAAATATTTCTGCACACATGGATTTTGGCGA
AGCCAGACTTGACAGCGTTACCATCAATGGTAACACATCCTATTGTGTCACTAAGCCTT
ACTTCAGGCTTGAAACTAACTTTATGTGTACAGGTTGCACTATGAATCTGCGCACTGAT
ACCTGTAGTTTTGACCTGTCAGCAGTAAACAATGGCATGTCATTCTCTCAATTCTGTCTA
AGCACTGAATCTGGTGCTTGTGAGATGAAAATTATTGTTACCTACGTATGGAATTACTTG
CTAAGGCAGCGTTTGTATGTTACAGCTGTAGAGGGCCAGACTCACACTGGAACCACTT
CAGTACATGCAACAGACACTTCTAGTGTAATCACTGATGTCTGCACTGATTACACTATCT
ATGGAGTCTCTGGTACTGGCATTATTAAGCCATCAGATCTCTTATTACATAATGGCATAG
CATTCACCTCTCCAACAGGTGAGCTCTATGCATTTAAAAATATAACCACTGGCAAAACC
CTTCAGGTCTTACCGTGTGAAACCCCTTCTCTACTGATTGTGATAAACAACACCGTTGT
CGGTGCTATCACATCCAGTAACTCAACTGAAAATAATAGGTTTACTACTACTATTGTCAC
ACCTACTTTCTTTTATTCCACAAATGCCACCACCTTCAACTGCACCAAGCCTGTTTTGT
CCTATGGACCCATCAGCGTGTGTAGTGATGGTGCAATTGCGGGAACATCCACATTACAG
AATACTCGACCATCCATAGTTTCACTATACGATGGCGAAGTTGAAATACCATCTGCATTT
TCTCTTTCTGTTCAGACGGAGTATTTGCAAGTTCAAGCAGAGCAAGTTATAGTTGATTG
TCCTCAGTATGTATGCAACGGCAACAGCCGTTGTCTACAATTACTGGCACAATACACCT
CAGCTTGCTCTAACATTGAAGCAGCTCTGCATTCCTCTGCACAGTTGGATAGCAGAGA
GATTATAAATATGTTTCAAACATCAACACAGTCCTTGCAGTTAGCTAATATTACCAACTT
CAAGGGTGACTACAATTTTAGCAGCATACTAACCACCAGACTAGGTGGCAGATCTGCTA
TTGAAGACCTTCTTTTTAATAAAGTTGTTACTAGTGGCCTTGGCACTGTTGATCAGGAC
TACAAAGCCTGCTCTAGAGACATGGCCATCGCTGACTTAGTTTGTTCCCAGTATTACAA
TGGCATCATGGTTCTACCTGGTGTTGTTGATGCTGAGAAAATGGCAATGTACACTGGCT
CTCTTACTGGAGCTATGGTATTTGGGGGACTGACTGCTGCAGCTGCAATACCCTTCGCT

ACAGCAGTACAAGCTCGCCTCAATTATGTCGCACTGCAAACAAATGTACTACAAGAAA
ACCAGAAAATTCTTGCAGAATCATTTAACCAAGCAGTTGGCAATATATCACTTGCACTA
TCTTCTGTTAATGATGCCATCCAGCAAACTTCTGAGGCTCTTAACACCGTAGCTATTGCT
ATTAAAAAGATTCAAACAGTTGTTAACCAGCAGGGTGAGGCATTATCACACCTGACTG
CACAGCTGTCAAACAATTTTCAAGCAATTTCGACTTCTATTCAAGACATTTACAACCGT
CTTGAGGAAGTAGAGGCTAACCAGCAAGTTGACCGTCTCATCACAGGACGGTTGGCT
GCACTTAATGCATATGTTACTCAGTTACTCAATCAGATGTCTCAGATTAGACAATCTCGA
TTGTTAGCTCAGCAAAAGATTAATGAGTGTGTCAAATCTCAGTCATCCAGATACGGTTT
CTGTGGAAATGGCACACACATCTTCTCACTTACACAGACTGCACCAAATGGCATATTTT
TCATGCATGCAGTGCTTGTACCCAACAAATTCACACGTGTCAACGCTTCTGCCGGCATT
TGTGTGGATAATATCAAAGGCTACTCATTGCAGCCTCAACTTATACTCTACCAGTTTAAT
AACTCCTGGAGAGTTACACCTAGAAATATGTATGAACCCAGACTGCCCCGGCAAGCTG
ATTTCATACAATTAACTGATTGCAGCGTTACTTTTTATAACACCACCGCTGCTAATCTTC
CCAATATTATCCCTGACATTATAGATGTCAATCAAACAGTCAGTGATATTATTGACAATTT
ACCTACAGCAACACCTCCTCAGTGGGATGTTGGTATCTATAACAACACTATTCTCAACC
TCACCGTTGAGATTAATGATCTACAAGAGCGGTCTAAAAACCTCTCACAGATTGCAGAT
CGTTTACAAAATTATATTGACAATCTTAACAATACTCTAGTTGACCTTGAATGGCTCAAC
AGAGTAGAAACTTACCTTAAATGGCCGTGGTATATATGGCTTGCCATTGCCCTGGCTCTT
ATTGCATTTGTGACAATCCTCATAACAATCTTTCTTTGTACTGGTTGTTGTGGTGGTTGC
TTTGGTTGTTGTGGCGGTTGTTTTGGCCTTTTCTCTAAGAAGAAAAGGTATACCGACGA
CCAACCAACACCGTCCTTTAAGTTTAAGGAATGGTAG

S gene sequence of ferrets' ileum tissue from 10 dpi
ATGCAGAGAGCTCTATTGATTATGACCTTACTTTGTCTCGTTCGAGCAAAGTTTGCTGAT
GATCTACTCGATTTGCTCACCTTCCCGGGTGCACATCGCTTCTTACATAAACTCACGAGT
AATTCCAGCAGTCTCTACTCGCGGGCTAATAACTTTGATGTTGGCGTTCTTCCTGGCTA
CCCCACTAAGAACGTTAACCTCTTCTCACCACTTACTAACTCTACTTTGCCAATTAATGG
CCTTCATCGGAGTTACCAACCTCTTATGCTGAATTGTCTTACTAAAATAACTAACCACAC
TCTCAGCATGTATCTCCTACCTAGTGAGATACAAACTTATAGCTGCGGCGGTGCCATGG
TTAAATACCAGACACATGATGCAGTTCGTATCATTTTAGACCTCACTGTCACTGACCAC
ATCTCTGTTGAAGTCGTTGGCCAACGTGGTGAAAATTATGTGTTTGTTTGTAGTGAGCA
GTTTAACTATACCACTGCATTACCCAACTCTACCTTCTTCTCACTTAATTCTGAGCTTTAT
TGCTTTATTAATAACACCTACTTAGGTATTCTTCCACCTGATTTAACTGACTTTACGGTCT
ATCGTACTGGGCAGTTTTATGCTAATGGTTACCTTTTAGGTACTTTACCTATTACGGTTAA
CTATGTAAGGTTGTATCGGGGTCATTTGTCGGCCAATAGTGCCCACTTTGCCCTTGCAA
ACCTAACCGATACACTCATAACACTTACCAATACTACTATATCGCAAATCACTTATTGTG
ATAAGTCAGTAGTTGATTCAATAGCATGCCAGCGCTCTTCTCACGAAGTGGAGGATGGG
TTTTACTCCGACCCTAAATCTGCCGTTAGAGCTAGGCAACGTACTATTGTTACACTACCT
AAGCTCCCTGAGCTTGAAGTAGTGCAGTTAAATATTTCTGCACACATGGATTTTGGCGA
AGCCAGACTTGACAGCGTTACCATCAATGGTAACACATCCTATTGTGTCACTAAGCCTT
ACTTCAGGCTTGAAACTAACTTTATGTGTACAGGTTGCACTATGAATCTGCGCACTGAT
ACCTGTAGTTTTGACCTGTCAGCAGTAAACAATGGCATGTCATTCTCTCAATTCTGTCTA
AGCACTGAATCTGGTGCTTGTGAGATGAAAATTATTGTTACCTACGTATGGAATTACTTG
CTAAGGCAGCGTTTGTATGTTACAGCTGTAGAGGGCCAGACTCACACTGGAACCACTT

CAGTACATGCAACAGACACTTCTAGTGTAATCACTGATGTCTGCACTGATTACACTATCT
ATGGAGTCTCTGGTACTGGCATTATTAAGCCATCAGATCTCTTATTACATAATGGCATAG
CATTCACCTCTCCAACAGGTGAGCTCTATGCATTTAAAAATATAACCACTGGCAAAACC
CTTCAGGTCTTACCGTGTGAAACCCCTTCTCTACTGATTGTGATAAACAACACCGTTGT
CGGTGCTATCACATCCAGTAACTCAACTGAAAATAATAGGTTTACTACTACTATTGTCAC
ACCTACTTTCTTTTATTCCACAAATGCCACCACCTTCAACTGCACCAAGCCTGTTTTGT
CCTATGGACCCATCAGCGTGTGTAGTGATGGTGCAATTGCGGGAACATCCACATTACAG
AATACTCGACCATCCATAGTTTCACTATACGATGGCGAAGTTGAAATACCATCTGCATTT
TCTCTTTCTGTTCAGACGGAGTATTTGCAAGTTCAAGCAGAGCAAGTTATAGTTGATTG
TCCTCAGTATGTATGCAACGGCAACAGCCGTTGTCTACAATTACTGGCACAATACACCT
CAGCTTGCTCTAACATTGAAGCAGCTCTGCATTCCTCTGCACAGTTGGATAGCAGAGA
GATTATAAATATGTTTCAAACATCAACACAGTCCTTGCAGTTAGCTAATATTACCAACTT
CAAGGGTGACTACAATTTTAGCAGCATACTAACCACCAGACTAGGTGGCAGATCTGCTA
TTGAAGACCTTCTTTTTAATAAAGTTGTTACTAGTGGCCTTGGCACTGTTGATCAGGAC
TACAAAGCCTGCTCTAGAGACATGGCCATCGCTGACTTAGTTTGTTCCCAGTATTACAA
TGGCATCATGGTTCTACCTGGTGTTGTTGATGCTGAGAAAATGGCAATGTACACTGGCT
CTCTTACTGGAGCTATGGTATTTGGGGGACTGACTGCTGCAGCTGCAATACCCTTCGCT
ACAGCAGTACAAGCTCGCCTCAATTATGTCGCACTGCAAACAAATGTACTACAAGAAA
ACCAGAAAATTCTTGCAGAATCATTTAACCAAGCAGTTGGCAATATATCACTTGCACTA
TCTTCTGTTAATGATGCCATCCAGCAAACTTCTGAGGCTCTTAACACCGTAGCTATTGCT
ATTAAAAAGATTCAAACAGTTGTTAACCAGCAGGGTGAGGCATTATCACACCTGACTG
CACAGCTGTCAAACAATTTTCAAGCAATTTCGACTTCTATTCAAGACATTTACAACCGT
CTTGAGGAAGTAGAGGCTAACCAGCAAGTTGACCGTCTCATCACAGGACGGTTGGCT
GCACTTAATGCATATGTTACTCAGTTACTCAATCAGATGTCTCAGATTAGACAATCTCGA
TTGTTAGCTCAGCAAAAGATTAATGAGTGTGTCAAATCTCAGTCATCCAGATACGGTTT
CTGTGGAAATGGCACACACATCTTCTCACTTACACAGACTGCACCAAATGGCATATTTT
TCATGCATGCAGTGCTTGTACCCAACAAATTCACACGTGTCAACGCTTCTGCCGGCATT
TGTGTGGATAATATCAAAGGCTACTCATTGCAGCCTCAACTTATACTCTACCAGTTTAAT
AACTCCTGGAGAGTTACACCTAGAAATATGTATGAACCCAGACTGCCCCGGCAAGCTG
ATTTCATACAATTAACTGATTGCAGCGTTACTTTTTATAACACCACCGCTGCTAATCTTC
CCAATATTATCCCTGACATTATAGATGTCAATCAAACAGTCAGTGATATTATTGACAATTT
ACCTACAGCAACACCTCCTCAGTGGGATGTTGGTATCTATAACAACACTATTCTCAACC
TCACCGTTGAGATTAATGATCTACAAGAGCGGTCTAAAAACCTCTCACAGATTGCAGAT
CGTTTACAAAATTATATTGACAATCTTAACAATACTCTAGTTGACCTTGAATGGCTCAAC
AGAGTAGAAACTTACCTTAAATGGCCGTGGTATATATGGCTTGCCATTGCCCTGGCTCTT
ATTGCATTTGTGACAATCCTCATAACAATCTTTCTTTGTACTGGTTGTTGTGGTGGTTGC
TTTGGTTGTTGTGGCGGTTGTTTTGGCCTTTTCTCTAAGAAGAAAAGGTATACCGACGA
CCAACCAACACCGTCCTTTAAGTTTAAGGAATGGTAG
